# Soluble Factors on Stage to Direct Mesenchymal Stem Cells Fate

**DOI:** 10.3389/fbioe.2017.00032

**Published:** 2017-05-17

**Authors:** Cristina Sobacchi, Eleonora Palagano, Anna Villa, Ciro Menale

**Affiliations:** ^1^Istituto di Ricerca Genetica e Biomedica (IRGB), Consiglio Nazionale delle Ricerche (CNR), Milan Unit, Milan, Italy; ^2^Human Genome Laboratory, Humanitas Clinical and Research Institute, Rozzano, Milan, Italy; ^3^Department of Medical Biotechnologies and Translational Medicine, University of Milan, Milan, Italy

**Keywords:** mesenchymal stem cells, growth factors, hormones, cytokines, RANKL, bone marrow microenvironment

## Abstract

Mesenchymal stem cells (MSCs) are multipotent stromal cells that are identified by *in vitro* plastic adherence, colony-forming capacity, expression of a panel of surface molecules, and ability to differentiate at least toward osteogenic, adipogenic, and chondrogenic lineages. They also produce trophic factors with immunomodulatory, proangiogenic, and antiapoptotic functions influencing the behavior of neighboring cells. On the other hand, a reciprocal regulation takes place; in fact, MSCs can be isolated from several tissues, and depending on the original microenvironment and the range of stimuli received from there, they can display differences in their essential characteristics. Here, we focus mainly on the bone tissue and how soluble factors, such as growth factors, cytokines, and hormones, present in this microenvironment can orchestrate bone marrow-derived MSCs fate. We also briefly describe the alteration of MSCs behavior in pathological settings such as hematological cancer, bone metastasis, and bone marrow failure syndromes. Overall, the possibility to modulate MSCs plasticity makes them an attractive tool for diverse applications of tissue regeneration in cell therapy. Therefore, the comprehensive understanding of the microenvironment characteristics and components better suited to obtain a specific MSCs response can be extremely useful for clinical use.

## Introduction

Great attention has been recently paid to the characterization and biomedical applications of multipotent adult stem cells present in the stromal compartment of several post-natal tissues, the “mesenchymal stem cells” (MSCs). MSCs, identified in bone tissue as precursor cells of osteoblasts\osteocytes, chondrocytes, and marrow adipocytes, are defined as multipotent cells that can be easily isolated from the stromal fraction. MSCs exhibit *in vitro* plastic adherence, fibroblast spindle-like shaped morphology, and expression of a panel of surface molecules that is continuously refined to identify unique markers for *bona fide* MSCs definition (Bourin et al., 2013; Schena et al., 2017). MSCs possess self-renewal and clonogenic capacity and highly proliferate and differentiate at least toward the osteogenic, adipogenic, and chondrogenic lineages both *in vitro*, by means of specific differentiation media, and *in vivo* in an ectopic bone formation assay (Schena et al., 2017). In bone, MSCs are located around sinusoids and along the perivascular network in the stroma (Sacchetti et al., 2007; Mendez-Ferrer et al., 2010), where they take part in the generation of the complex and heterogeneous system of the bone marrow microenviroment (BM-ME). In fact, MSCs together with pericytes, adventitial cells, endothelial cells, fibroblasts, marrow adipocytes, and hematopoietic and immune cells generate a dynamic compartment by establishing cell-to-cell interactions and producing soluble factors with autocrine and paracrine functions (Moore and Lemischka, 2006; Bianco et al., 2013). Many reports in literature deal with the MSCs’ secretome, i.e., the variety of factors released by MSCs in physiopathological conditions. For example, MSCs exert immunomodulatory properties on innate and adaptive immune cells by sensing inflammatory environments (Bernardo and Fibbe, 2013) and secreting pro- and anti-inflammatory chemokines (Keating, 2012; Le Blanc and Mougiakakos, 2012).

Moreover, MSCs organize the vascular network, since they interact with endothelial and hematopoietic cells by producing or responding to different molecules (e.g., VEGF, FGF-2, PDGF-α, and TGF-β1) (Jain, 2003; Sacchetti et al., 2007) and synthesize antiapoptotic factors (e.g., HGF and IGF1) in pathological conditions (Nagaya et al., 2005; Kennelly et al., 2016). MSCs also exert supportive functions for hematopoietic stem cells (HSC), thanks to direct cell-to-cell contact and secreted trophic molecules, e.g., jagged 1 and BMPs (Calvi et al., 2003; Zhang et al., 2003; He et al., 2017). Furthermore, they modulate osteoclast formation, survival, and resorptive activity through positive and negative regulatory molecules, among which RANKL and OPG are the iconic ones (Sharaf-Eldin et al., 2016). Finally, MSCs differentiation and secretory activities are relevant in skeletal pathologies such as multiple myeloma (MM), bone metastases, and bone marrow failure syndromes (BMFS), and their capacity to support and/or regulate hematopoiesis and cancer cells survival has been extensively described (Mundy, 2002; Kassen et al., 2014; David Roodman and Silbermann, 2015; Fairfield et al., 2016).

From the opposite perspective, neighboring cells or cells residing in other tissues in turn provide stimuli influencing MSCs properties in physiopathological conditions.

Here, we exactly aim to take this latter point of view and to provide some examples of soluble factors present in BM-ME that are able to direct MSCs fate and orchestrate their cellular response. MSCs secretome (Murphy et al., 2013) and plasticity make them an attractive tool for biomedical applications, such as tissue regeneration and cell-based therapy for several diseases. The capacity to modulate functional properties of MSCs is essential for their optimal exploitation in clinical practice. To this final goal, a wider understanding of the variety of molecular and cellular interactions in BM-ME is of paramount importance.

## Microenvironment Factors Orchestrate MSCs Fate

**Figure 1:**
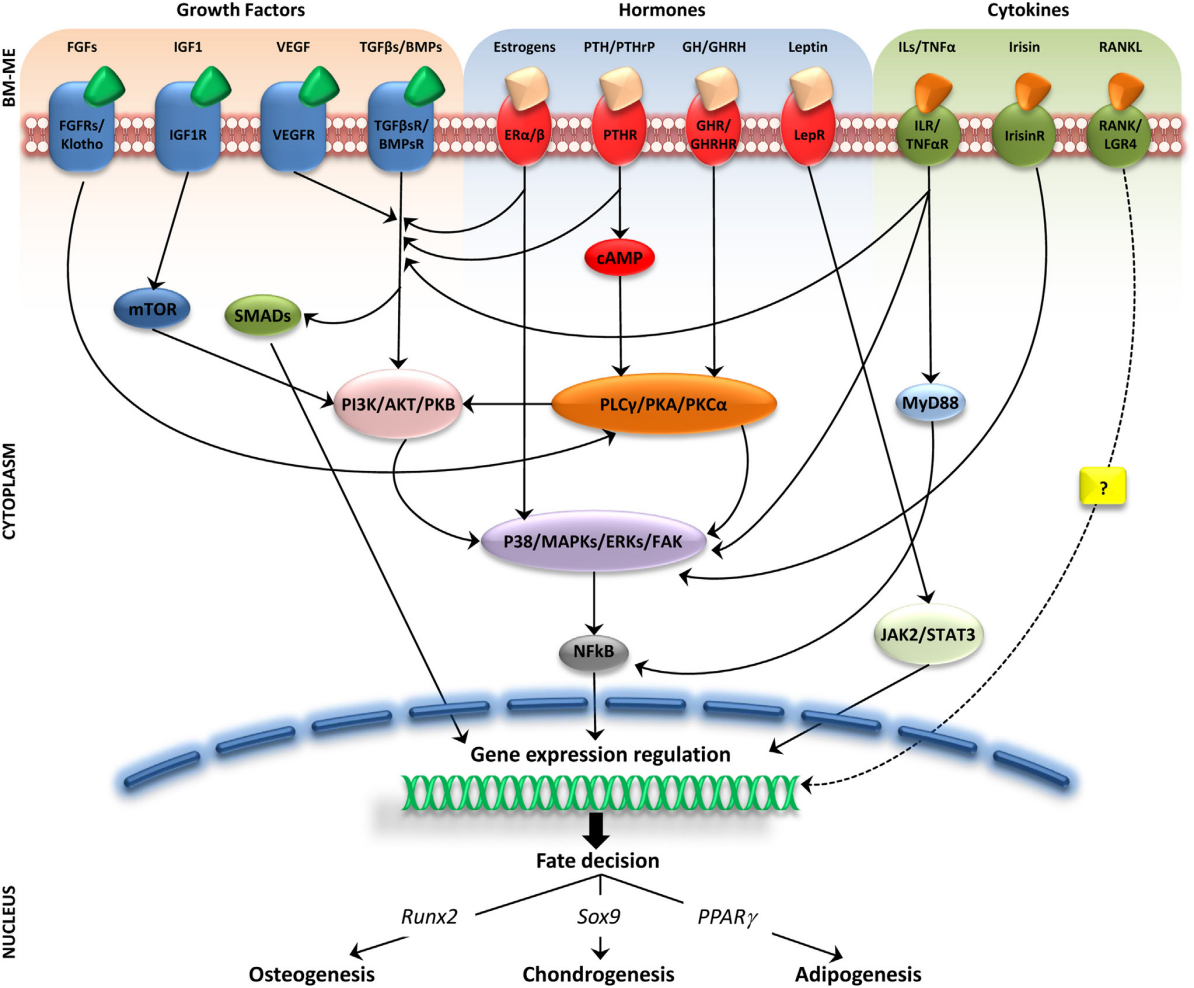
**Molecular pathways activated by soluble factors influencing bone marrow mesenchymal stem cells (MSCs) differentiation**. Simplified representation of cellular players in bone marrow microenviroment (BM-ME) showing that growth factors, hormones, and cytokines, by binding to their respective receptors on the MSC plasma membrane, trigger activation of signaling cascades that ultimately result in gene expression regulation relevant for MSCs differentiation fate.

**Figure 2:**
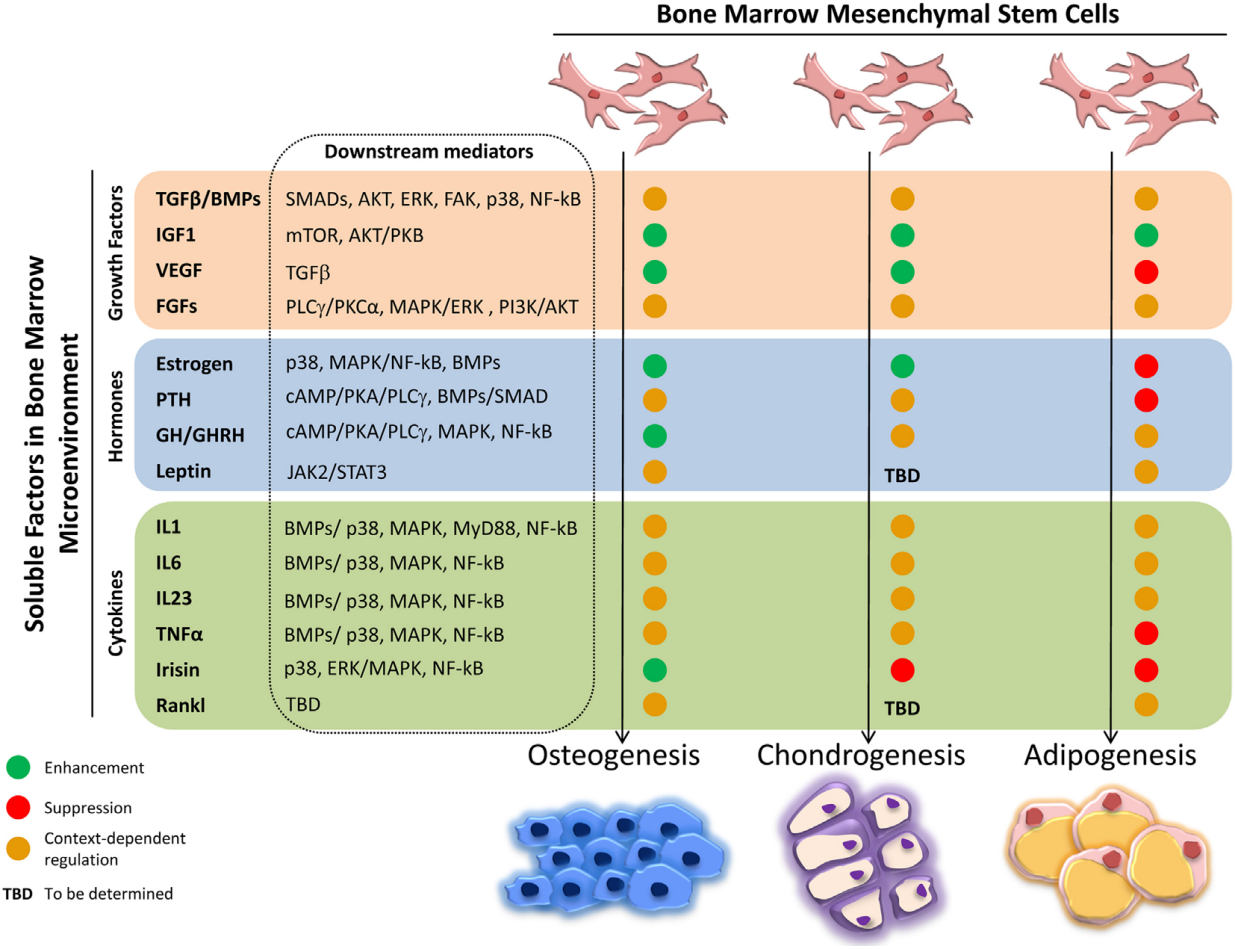
**Effects of soluble factors on bone marrow mesenchymal stem cells (MSCs) trilineage differentiation**. Schematic representation of the effect exerted by the described soluble molecules on MSCs osteogenic, chondrogenic, and adipogenic differentiation. Green circle, enhancement; red circle, suppression; orange circle, context-dependent regulation; TBD, to be determined.

The intense cellular interactions in the BM make this microenvironment a dynamic compartment where several soluble factors are able to modulate MSC functions. Here, we will describe some of these molecules, their signaling pathways (Figure 1), and their final effect on MSC fate (Figure 2).

### Growth Factors (GFs)

In general, GFs influence MSCs acting both in a paracrine and autocrine manner; in fact, MSCs express on their surface GF receptors.

Proteins of the TGFβ/BMP superfamily are the most abundant GFs in BM-ME and originate mainly from bone matrix degradation and activated T cells (Tang et al., 2009; Croes et al., 2016; Li et al., 2016). The TGFβ family comprises three members: TGFβ1, TGFβ2, and TGFβ3. TGFβ1, released from the bone matrix by the osteoclast resorptive activity, has been demonstrated *in vitro* and *in vivo* to induce MSCs migration to the remodeling sites, thus coupling bone formation and resorption. The mechanism through which this function is exerted, whether through the canonical signaling pathway, involving SMADs, or the non-canonical one, involving AKT, ERK1/2, FAK, and p38, is debated (Tang et al., 2009; Dubon et al., 2017). Moreover, *in vitro* TGFβ1 drives MSCs fate toward osteoblasts generation and inhibits adipogenic differentiation; accordingly, TGFβ1 induces the switching from adipogenesis to osteogenesis when added to an adipogenic medium, acting mainly on the SMAD/C/EBPs/PPARγ signaling (Choy and Derynck, 2003; van Zoelen et al., 2016). In other reports, during *in vitro* MSC expansion, TGFβ1 reduces the number of osteoprogenitor cells and limits their expansion inducing a rapid terminal differentiation, suggesting that TGFβ1 effects on MSCs might depend on the commitment state of the cells (Walsh et al., 2003; Claros et al., 2014). Furthermore, TGFβ1 is a key molecule in chondrogenesis by stabilizing SOX9 *via* the canonical SMAD or the non-canonical p38 pathways (Coricor and Serra, 2016; Dexheimer et al., 2016). A similar potent effect on chondrogenic differentiation has been demonstrated for TGFβ2 and TGFβ3 (Vinatier et al., 2009).

Furthermore, TGFβ1 induces ADAM12 expression in MSCs/pericytes triggering myofibroblast transdifferentiation and contributing to fibrosis (Cipriani et al., 2016).

The BMP family comprises at least 15 members, which usually exert synergistic effects with TGFβs and activate SMAD transcription factors and expression of genes such as *Runx2, Pparγ*, or *Sox9*. This ultimately results in the promotion of MSCs differentiation toward the adipogenic (e.g., BMP2, BMP4, and BMP7), osteogenic (e.g., BMP2, BMP6, and BMP9), or chondrogenic (e.g., BMP2 and BMP7) lineage, depending on the microenvironmental concentrations (Kang et al., 2009; Chen et al., 2012).

IGF1, a polypeptide with a high-binding affinity to IGF1R and insulin-like features (Wang et al., 2013), is one of the most abundant GFs deposited in the bone matrix. *In vitro* and *in vivo* in mouse and rat models, IGF1 released from bone matrix degradation induces osteoblast differentiation *via* the mTOR pathway and enhances osteoblasts function (Xian et al., 2012; Crane and Cao, 2014). Regarding chondrogenesis, IGF1 in combination with TGFβs enhances *in vitro* chondrocytes proliferation and collagen II production (Fukumoto et al., 2003; Indrawattana et al., 2004), while for the adipogenic fate commitment, IGF1 activates IGF1R-dependent AKT/PKB signaling, increasing *Pparγ* expression and lipid accumulation (Scavo et al., 2004).

VEGF is a key soluble molecule involved in endothelial cell proliferation, migration, and tissue vascularization (Ferrara et al., 2003). MSCs/osteoblasts themselves express VEGF and its receptors, so this GF may exert both a paracrine and an autocrine regulation (Niida et al., 1999; Nakagawa et al., 2000; Kaigler et al., 2003; Yang et al., 2008; Liu and Olsen, 2014; Marsano et al., 2016). VEGF is essential for coupling angiogenesis to bone formation during skeletal development, by promoting chondrocytes survival in hypoxic regions of cartilaginous templates, vascularization of developing bones, and proliferation and differentiation of osteoblasts (Maes et al., 2004; Zelzer et al., 2004). In post-natal bone homeostasis, VEGF favors MSCs osteoblastogenesis at the expense of adipogenesis, through intracrine regulation of *Runx2* and *Pparγ* (Liu et al., 2012), while intracellular blockade of VEGF signaling in MSCs activates TGFβ signaling, thus inducing spontaneous *in vivo* chondrogenesis and formation of a hypoxic microenvironment and a stable hyaline cartilage (Marsano et al., 2016).

In FGF family, many members positively regulate MSCs proliferation and osteogenic differentiation, by interacting with FGFR2 and activating PLCγ/PKCα, MAPK/ERK 1/2, and PI3K/AKT pathways (Marie, 2012). FGFs are also crucial for the regulation of MSCs chondrogenic differentiation through FGF/FGFR3, as demonstrated by their involvement in the pathogenesis of different forms of chondrodysplasia (Ornitz and Legeai-Mallet, 2017). However, also for these GFs, results in literature are discordant: recent data indicate that FGF1 and FGF2 maintain MSCs in an uncommitted state, preventing their differentiation (Le Blanc et al., 2015; Simann et al., 2017). Finally, the hormone-like FGF23, produced by bone cells and by cells of different tissues, favors osteogenic differentiation at the expenses of adipogenesis by binding to its receptor Klotho, which mediates the activation of MAPKs signaling (Li et al., 2013c).

### Hormones

The skeleton is widely recognized as both an endocrine organ and a target for other endocrine tissues (Fukumoto and Martin, 2009).

The prototypical example of MSCs-regulating hormone is estrogens, the main molecules involved in post-menopausal osteoporosis. Estrogens bind their α and/or β receptors and induce MSCs proliferation and osteogenic and chondrogenic differentiation (Rodriguez et al., 2008), through the activation of BMPs/WNT/β-catenin and p38 MAPKs/NF-κB signaling pathways (Gopalakrishnan et al., 2006; Li et al., 2013b; Kim et al., 2015; Cong et al., 2016). Moreover, estrogens induce early osteoblast differentiation and inhibit adipogenesis in mice (Okazaki et al., 2002). Furthermore, they reduce LPL levels, impair adipocyte progression into hypertrophic state, and influence body adipose tissue depots distribution and glucose metabolism (Post et al., 2008). Overall, this evidence points to a role of estrogens in regulating both bone and glucose homeostasis.

PTH is one of the principal modulator of calcium homeostasis through cAMP/PKA/PLCγ signaling. It also displays both catabolic and anabolic functions in bone remodeling: the former is exerted by inducing RANKL production, which fosters osteoclasts’ generation and activity, the latter by affecting MSCs fate (Hock and Gera, 1992; Qin et al., 2004). Indeed, PTH induces *in vitro* osteogenic differentiation *via* LRP6-dependent BMP/SMAD signaling (Polo and Di Fiore, 2006; Jilka, 2007; Qiu et al., 2010; Yu et al., 2012). Accordingly, *in vivo* deletion of PTHR in murine MSCs reduces bone formation and increases bone resorption and marrow adiposity, while intermittent PTH administration to control mice reduces marrow adipogenesis (Fan et al., 2017). On the contrary, PTHrP affects MSCs differentiation, preventing chondrocyte hypertrophy and blocking osteogenesis through regulation of *Sox9* and *Runx2* gene expressions (Provot et al., 2006; Zhang et al., 2009; Fischer et al., 2014).

GH regulates linear growth during development (Gomes et al., 2013; Ma et al., 2016) and is involved in BM adiposity maintenance. It enhances adipocytes and osteoblast precursor pool size, while it induces MSCs osteogenesis and inhibits BM fat accumulation (Menagh et al., 2010). Its regulator, GHRH, has receptors (GHRHR) also on MSCs (Gomes et al., 2013; Ma et al., 2016) and through their binding promotes MSCs proliferation and survival *via* the cAMP/PKA/PLCγ signaling, activates MAPK signals, and induces osteogenic differentiation (Jaiswal et al., 2000; Xia et al., 2016).

Finally, the adipose tissue-derived hormone leptin contributes to guide MSCs commitment but contradictory results are reported (Ducy et al., 2000; Kontogianni et al., 2004; La Cava and Matarese, 2004). MSCs highly express leptin receptor (Zhou et al., 2014), and *in vitro*, leptin enhances osteogenic differentiation and reduces the adipogenic one (Thomas, 2004). On the contrary, leptin *in vivo* regulates MSCs, increasing marrow adipogenesis and reducing osteogenesis in response to diet and adiposity, through the JAK2/STAT3 pathway (Yue et al., 2016).

### Cytokines

Among the cell populations present in BM-ME, immune cells participate in directing MSCs fate by secreting a variety of cytokines, with anabolic or anti-anabolic effects depending on the inflammatory state of the bone tissue.

For example, T cells activate bone formation by producing Wnt ligands that initiate Wnt signaling and osteoblastogenesis (Ouji et al., 2006; Terauchi et al., 2009). They also produce CD40L that binds CD40 on MSCs inducing their proliferation and survival (Ahuja et al., 2003; Gao et al., 2008; Li et al., 2013a).

Conflicting results are reported regarding the effects of proinflammatory cytokine on MSCs differentiation. In a proinflammatory environment, the interleukins IL-1β, IL-6, and IL-23 (mainly derived from Th17 cells) have been reported to increase the differentiation performance of human MSC toward the osteogenic and adipogenic lineages (Pourgholaminejad et al., 2016). Accordingly, TNFα, IL-1β, and IL-6 enhance osteoblast differentiation by triggering NF-κB signaling or modulating BMP2 pathway (Nakase et al., 1997; Hess et al., 2009; Huh and Lee, 2013; Croes et al., 2015).

On the contrary, IL-1 and TNFα inhibit MSCs osteogenesis and adipocyte generation, acting not only through the canonical NF-κB signaling (Lacey et al., 2009; Sullivan et al., 2014) but also *via* other mechanisms such as IL-1R1/MyD88 signal transduction, as described for murine MSCs obtained from different genetic backgrounds (Martino et al., 2016). Similarly, IL-6 can impair MSCs ability to generate adipocytes and chondrocytes and keep them in an undifferentiated state by activating ERK1/2 (Pricola et al., 2009).

Recently, great attention has been paid to the skeletal muscle-derived myokine Irisin, which is able to directly target the bone tissue, thus regulating its physiology. Specifically, Irisin can induce MSCs osteoblast differentiation through p38/ERK MAPK signaling pathways, leading to the upregulation of osteogenic marker genes, such as *Atf4, Runx2, Osx, Lrp5, β-catenin, Alp*, and *Col1a1* (Colaianni et al., 2015; Qiao et al., 2016).

#### RANKL Involvement in MSCs Fate Decision

RANKL is the essential osteoclastogenic factor produced mainly by MSCs, osteoblasts, and osteocytes (Sobacchi et al., 2007, 2013; Nakashima et al., 2011) and also by T cells in the bone marrow (Pacifici, 2016a,b). The possibility that RANKL might be an additional factor in BM-ME influencing MSCs properties has been considered only lately. In fact, recent reports indicate that RANKL might have bone anabolic effects when pulsed or low doses of the cytokine are administered to ovariectomized mice (Buchwald et al., 2015; Cline-Smith et al., 2016). In line with these observations, our group has found that BM-MSCs derived from RANKL-deficient mice display a partial osteogenic differentiation defect, which is improved by restoring the production of the soluble form of the cytokine. Our data suggest that RANKL might contribute to direct MSCs fate in an autocrine/paracrine manner, likely through the interaction with either its receptor RANK (Schena et al., 2017) or the recently identified RANKL receptor LGR4 (Luo et al., 2016) (an R-spondin receptor, suggested to regulate bone formation in synergy with Wnt3a), which are both expressed in MSCs (Schena et al., 2017). On this basis, we might speculate that fine tuning, rather than completely blocking, RANKL could be relevant to regulate bone physiology.

## MSCs Fate in Pathological Conditions

### Multiple Myeloma

Multiple myeloma is a common hematological malignancy mainly characterized by osteolytic lesions due to increased osteoclast number and activity and strongly decreased bone formation (Kassen et al., 2014). MSCs and osteoblasts support MM cells survival, proliferation, and progression (Azab et al., 2009; Reagan et al., 2014; Roccaro et al., 2014; Fairfield et al., 2016), while osteogenic differentiation is reduced in MM patients, which might be a putative strategy of MM cells to preserve cells (e.g., MSCs) necessary for their support (Corre et al., 2007; Reagan et al., 2014). Cell-to-cell contact and production of soluble factors are likely involved in these mechanisms. For example, MM cells inhibit *Runx2* and inactivate the non-canonical Wnt5a/Ror2 pathway; a putative role of IL-7 produced by MM cells can be hypothesized (Giuliani et al., 2005; D’Souza et al., 2011; Bolzoni et al., 2013). Moreover, MM cells secrete Wnt inhibitory factors, i.e., Dkk1 and sclerostin; TGFβ, which impairs osteoblast differentiation (Lee et al., 2003; Tian et al., 2003; Colucci et al., 2011); and also factors inducing MSCs growth that, in turn, produce osteoclast-activating factors (i.e., IL-6, MCSF, TNFα, and RANKL) leading to osteolysis (David Roodman and Silbermann, 2015).

### Breast Cancer (BC) and Prostate Cancer (PC)

Breast cancer cells preferably metastasize to bone inducing purely osteolytic lesions, *via* the production of osteoclast-activating factors (mainly RANKL and MCSF). Furthermore, osteolytic lesions are production sites of several soluble factors derived from osteoclasts’ resorption of the bone matrix, such as TGFβ (Kang et al., 2003). These molecules can inhibit osteoblast development and functions and are able to induce BC cell proliferation and progression that, in turn, sustain the secretion of osteoblast inhibitory factors (Mundy, 2002).

Bone metastases in PC tend to be osteosclerotic, rather than osteolytic. PC cells produce soluble factors, e.g., BMPs, TGFβ, IGF1, FGFs, and VEGF, which increase MSCs osteogenic differentiation, osteoblast development, and bone deposition, leading to elevated mineral apposition, even though the newly formed bone is immature and of poor quality (Guise et al., 2006; David Roodman and Silbermann, 2015). Of note, the osteoclast-inducing hormone PTHrP, above reported as osteogenic inhibitor, is highly produced by PC cells and in this context enhances osteoblast progenitors’ proliferation and early osteogenesis (Liao et al., 2008).

### Bone Marrow Failure Syndromes

Bone marrow failure syndromes are hematological disorders characterized by impaired hematopoiesis comprising different phenotypes, i.e., myelodysplastic syndromes (MDS), aplastic anemia (AA), and chronic idiopathic neutropenia (CIN). MSCs play an important role in maintaining and restoring hematopoiesis, thanks to the secretion of regulatory factors for HSC functionality (Kastrinaki et al., 2013). Scanty data are available the other way round. For example in AA, MSCs osteogenic capacity is inhibited in favor of adipogenesis (Papadaki et al., 2001; Shipounova et al., 2009; Xu et al., 2009). Recently, oncostatin M, a member of the IL-6 family, has been reported to stimulate HSC expansion, inhibiting adipogenic differentiation and enhancing osteogenic differentiation of MSCs. Upon administration in mice bearing BM injury, it decreases marrow adipogenesis and restores HSC number (Sato et al., 2014).

## Conclusion

Bone marrow microenviroment is constituted by many diverse cell types, which establish an intense cross-talk among them. In particular, in recent years, MSCs have gained great attention for their trophic support to other cells, ability to secrete bioactive factors and plasticity, and for the possibility to be exploited in regenerative medicine applications. On the other hand, the outcome of much experimentation has failed to meet the forecasted expectations. The real challenge that still has to be faced is the global understanding of the cellular and molecular mechanisms, which take place in BM-ME and star MSCs as main character or as target.

This review gives just a flavor of the variety of soluble factors provided by neighboring cells, by the ECM or by other tissues influencing MSCs properties in pathophysiological settings. Many of these factors may elicit opposite MSCs behavior depending on the overall environmental conditions, as demonstrated by the controversial results reported in literature. Furthermore, the signaling pathways activated downstream each ligand/receptor interaction often intersect and then intertwine or diverge, thus generating an additional layer of complexity. The recent highlight on RANKL as a putative novel regulator of MSCs fate raises the possibility that additional factors involved in orchestrating MSCs functions have still to be recognized. A wider landscape of molecular and cellular interactions and of rules to be accomplished or modified to elicit specific cell behaviors needs to be reached. This deep understanding will improve the capacity to manipulate BM-ME and to effectively use MSCs for cell therapy.

## Author Contributions

All the authors contributed to organize, draft, and revise the manuscript.

## Conflict of Interest Statement

The authors declare that the research was conducted in the absence of any commercial or financial relationships that could be construed as a potential conflict of interest.
